# Deep Venous Reflux Associated with a Dilated Popliteal Fossa Vein Reversed with Endovenous Laser Ablation and Sclerotherapy

**DOI:** 10.1155/2013/242167

**Published:** 2013-10-23

**Authors:** Daniel P. Link, Jennifer Feneis, John Carson

**Affiliations:** Department of Vascular Surgery, University of California Davis School of Medicine, 4860 Y Street, STE 3400, Sacramento, CA 95817, USA

## Abstract

*Objective*. To report an incidence of reflux in the deep venous system reversed by ablation of a popliteal fossa vein (PFV). *Method*. A 40-year-old man with pain and swelling in the medial upper calf was found to have an incompetent PFV. *Results*. Reflux in the femoral and popliteal veins was reversed utilizing endovenous laser ablation and foam sclerotherapy, documented on Duplex studies before and after the intervention. There was also resolution of symptoms. *Conclusion*. A PFV can be associated with deep venous reflux. Correction of this reflux with ablation of the PFV suggests that his type of reflux is secondary to volume effects of the incompetent popliteal vein.

## 1. Introduction

The popliteal fossa vein (PFV) has been described as a tributary of the popliteal vein found in the popliteal fossa, which is anatomically distinct from the great and small saphenous veins [1, 2]. Classified as a perforating vein at the precongress meeting of the Fourteenth World Congress of the International Union of Phlebology (IUP) in 2001, PFV perforates the muscular fascia to connect superficial veins with deep veins, namely, the popliteal vein [1–3]. The prevalence of reflux in a PFV has varied between 8% in patients with nonsaphenous venous reflux [1] and 1% in patients with primary varicose veins [4]. This case presents a patient with a dilated PFV, with associated symptomatic venous insufficiency and deep venous reflux, reversed with endovenous laser ablation (EVLT) and sclerotherapy.

## 2. Case Information

**Figure 1:**
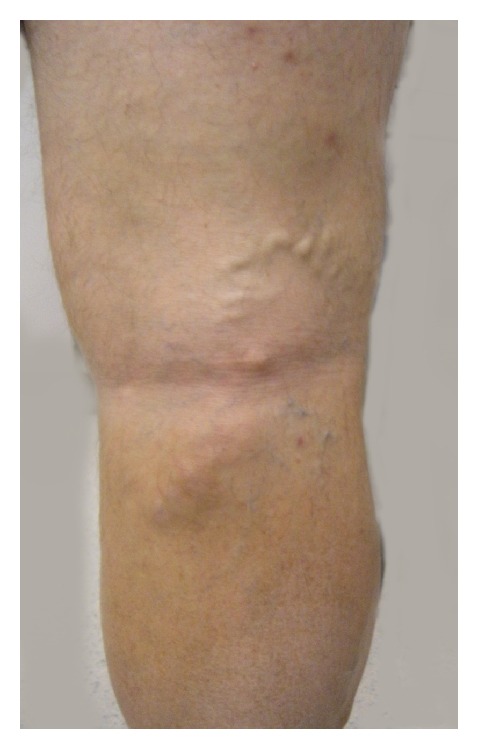
Photograph of leg demonstrating posterior popliteal varicosities and venous stasis pigmentation.

**Figure 2:**
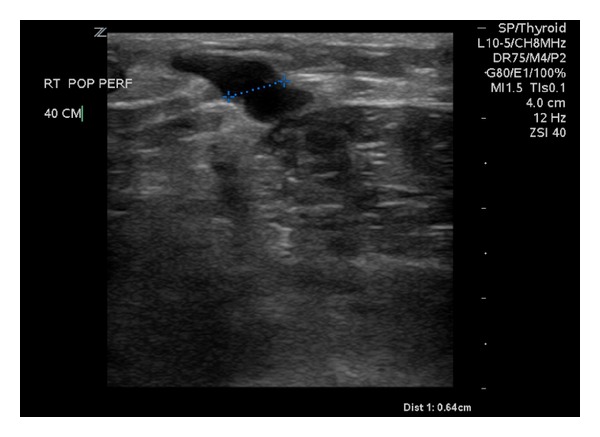
Popliteal fossa vein entering the deep fascia 40 cm from the medial malleolus, 6.4 mm at the fascial plane.

**Figure 3:**
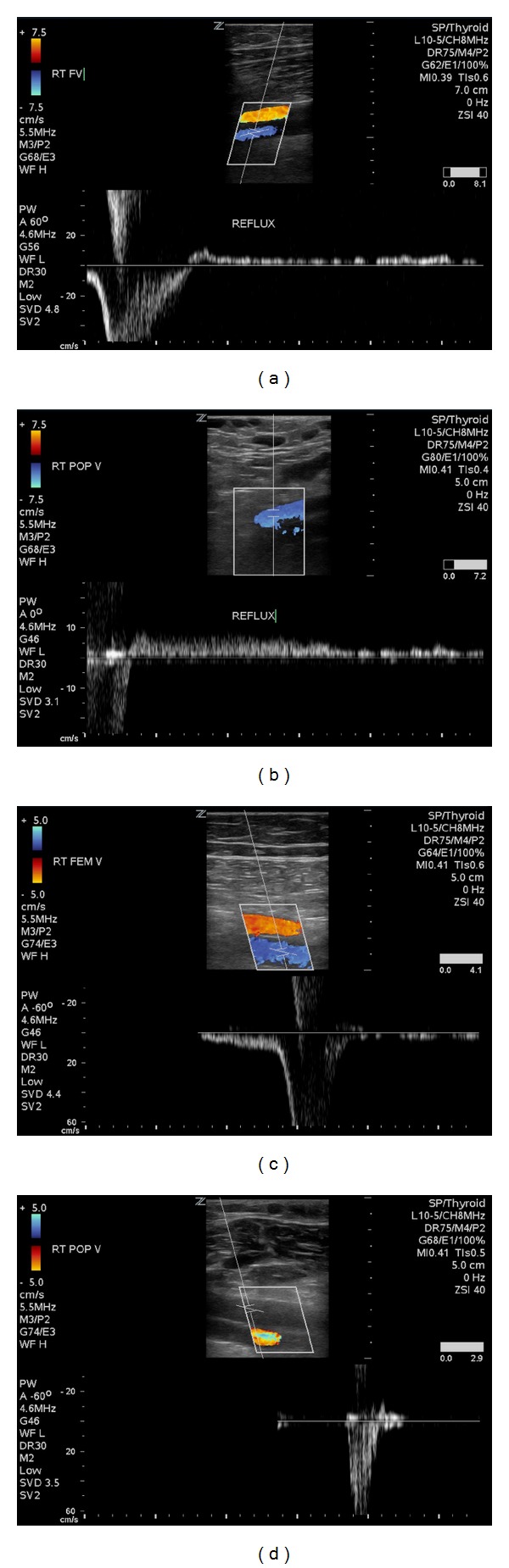
Pre procedure Duplex of the right femoral v. (a) and right popliteal v. (b); Post procedure Duplex of the right femoral v. (c) and right popliteal v. (d).

A 40-year-old man presented to the Vascular Center Clinic with symptomatic chronic venous insufficiency (CVI). The patient began experiencing cramping and swelling in his right leg seven years prior, with worsening symptoms over the past two years. Upon initial physical exam, the patient was noted to have 1+ edema, hyperpigmentation in the “gaiter zone” distribution, and varicosities on the posterior and medial aspect of the right knee (C4a) [5] (VCSS: skin pigmentation 2, inflammation 1, induration 2, active ulceration 0, and compression 3) [6, 7], as shown in Figure 1 [5]. After 14 weeks of compression therapy with thigh-high compression hose, the patient returned to clinic with no significant change in symptoms. Physical exam at the time of the followup revealed a mild increase in the venous stasis pigmentation of the right gaiter zone, with no associated edema or ulceration. Venous color duplex ultrasonography of the right lower extremity at this visit revealed reflux in the common femoral, femoral and popliteal veins. The degree of reflux increased dramatically to the popliteal vein, Figures 3(a) and 3(b). The reflux in the popliteal vein and femoral vein was greater than 5 sec standing with augmentation. The velocities were higher in the popliteal vein. Our laboratory considers reflux greater than 5 seconds significant under these conditions. A large perforating vein from the popliteal fossa, demonstrated in Figure 2, connected the dilated subdermal veins in the medial calf to the popliteal vein approximately 40 cm superior to the medial malleolus (6.4 mm at the fascial plane). EVLT of the large popliteal fossa vein was performed; 432 joules at 14 watts were applied over a 3 cm section of vein using an EVLT Perforator Vein Ablation Kit (Diomed Inc., Andover, MA, USA). Foam sclerotherapy with 2 ml of 0.5% sodium tetradecyl sulfate (AngioDynamics, NY, USA) of the large mid-calf varicosities immediately followed the EVLT. The treatment resulted in resolution of the patient's right leg pain and reflux in the common femoral vein. Two month followup demonstrated near complete resolution of the reflux in the femoral and popliteal veins on repeat Duplex scan, as demonstrated in Figure 3. All terminology this report conforms with standards based on Caggiati et al. [3, 8].

## 3. Discussion

PFV reflux has been previously demonstrated to be associated with higher rates of proximal, distal, superficial, perforator, and complex pattern reflux as compared to limbs without PFV; however, the prevalence of deep reflux was not statistically different between the two groups [2]. Treatment of incompetent perforating veins has previously focused on using subfascial endoscopic perforating vein surgery (SEPS) and sclerotherapy [9–11].

In this case, an incompetent popliteal fossa vein led to a large complex of pressurized subdermal veins as well as reflux in the femoral and popliteal veins. It is likely that a proximal calf perforating vein such as a popliteal fossa vein would produce more pressure in the subdermal calf veins than similar sized more distal veins [12]. It is unclear if either EVLT or sclerotherapy alone could have resulted in the identical ideal outcome at 1 year after procedure. The addition of the sclerotherapy seemed appropriate to reduce the rate of recurrent symptoms [13]. Reflux in the deep veins was reversed with EVLT of the PFV, which implies that the valve leaflets of the deep veins were not permanently damaged. It is likely that the incompetent PFV first created the subdermal varicosities, and as the venous hypertension continued, the deep veins became engorged as well. This venous volume overload leads to dilatation of the deep veins, rendering their valves incapable of stopping the backflow of blood in the relaxed state. When EVLT successfully obliterated the incompetent PFV, it reduced the overflow into the deep venous system, decreasing the diameter of the deep veins and allowing their valves to return to a functional state. The valve leaflets must have been still intact, or the reflux would not have been able to be reversed. This condition, occurring with an incompetent PFV, may be more prevalent than previously reported. Once identified, it may be effectively treated with minimally invasive techniques.
